# Abstracts from the 6th International Symposium of Advanced Topics in Exercise Physiology: Interval training as an efficient strategy to overcome the XXI century diseases with emphasis in the mental brain diseases: Ensenada Baja California, México. 27-28 October 2022 (virtual symposium)

**DOI:** 10.1186/s12919-023-00251-4

**Published:** 2023-03-02

## A1 Lifestyles and their relationship with the psychological status of postgraduate students in the area of physical activity

### Abraham Martínez Sandoval, Rosa María Cruz Castruita

#### Facultad de Organización Deportiva, Universidad Autónoma de Nuevo León, Nuevo León, México

##### **Correspondence:** Rosa María Cruz Castruita, rosa.cruzcst@uanl.edu.mx

**Background:** During the university period, students may present some health problems, which are mainly associated with their lifestyles. Behavioral factors such as the practice of physical activity, eating habits, and sleep quality is conditioning factors to achieve a good state of health. Psychosocial risk factors are also determinants for mental health in young people, one of which is coping skills. Objective to evaluate the relationship between the level of physical activity with the psychological state, eating habits, and sleep quality of postgraduate students in the area of physical activity and sports assigned to a public university in the generations of years 2022 and 2023.

**Materials and methods:** it is proposed to carry out a quantitative study with a cross-sectional descriptive design, in a sample of 33 participants. Data will be collected with the International Physical Activity Questionnaire (IPAQ), the Eating Habits Survey, the Pittsburgh Sleep Quality Index (PSQI) Questionnaire, the DASS 21 Scale, and the Academic Stress Coping Scale (A-CEA).

**Results:** To date, a health campaign has been developed to promote physical activity, healthy lifestyles, and fitness strategies through the placement of physical posters in the faculty and digital infographics that will be published by the communication networks of the faculty on Monday, Wednesday, and Friday.

**Conclusions:** knowing the lifestyles of university students is essential, to have a representation of the state of physical and mental health and their lifestyles to carry out healthy strategies and actions to strengthen the application of initiatives such as promoting Universities.

## A2 Impact of a remote supervised moderate interval training on cognitive functions in Mexican older adults. A preliminary study

### Ermilo Canton Martinez^1^ , Iván Renteria^2^ , Rubén Avilés Reyes^2^ , Juan Pablo Machado Parra^2^, Patricia C. García-Suárez ^1,3^, Barbara Mello Antunes^1^, Alberto Jiménez Maldonado^1^

#### ^1^Facultad de Deportes, Universidad Autónoma de Baja California, México, ^2^Facultad de Ciencias Administrativas y Sociales, Universidad Autónoma de Baja California, México, ^3^Department of Health, Sports and Exercise Sciences, University of Kansas, Lawrence, KS, United States

##### **Correspondence:** Alberto Jiménez Maldonado, jimenez.alberto86@uabc.edu.mx

**Background**: The social distancing was one of the protocols rigorously applied throughout the world to contain the COVID-19 spread, this condition increased the sedentary lifestyle in all population. Consequently, the lack of physical activity practice increased the vulnerability to suffer mental disorders, moreover the sedentary lifestyle is *per se* a risk factor to decrease the performance in cognitive tasks. The current work was focused to assess the impact of moderate interval training (MIT) employing the remote supervision on cognitive functions in older adults during the COVID-19 quarantine.

**Materials and methods**: Twelve elderly Mexican (age:67.78±6.4yrs; education:14.6±3.2yrs) carry out the training. The protocol involved 48 sessions of 60 min with a frequency of 4/times/week. The exercises program (30 sec workout/ 30 sec of rest) implicated chairs stand, bicep curls, one-arm row, wall push-ups, leg extensions, standing heel raises, standing kickbacks, and abdominal crunches at moderate intensity (12-14 on the Borg Scale). The 30-sec chair stand test was employed to determine the lower body strength. Additionally, the visuo-constructional ability and visual memory the Rey–Osterrieth Complex Figure (ROCF) was applied. Finally, the cognitive function was measured with the Montreal Cognitive Assessment (MOCA) questionnaire.

**Results**: Short-term-memory improved with the MIT (14.0vs20.0 pt.), p=0.002. Likewise, the long-term-memory also was improved with the intervention (9.5vs14.0), p=0.002. The working memory was better after the exercise program (16.00 vs 18.50), p=0.18. Opposite, the depression levels were not modified by the training (5.0 vs 5.0), p=0.71. Finally, the interval training increased the lower body strength (14.7vs19.4rep) p=0. 0159.

**Conclusions**: The initial data of the current work indicate that the remote supervised interval training is a feasible intervention to improve the brain function linked with the cognition (short-and long-term memory) in elderly. Those results were not associated with changes in the

depression levels.

## A3 Bone diameters and their association with causes of health risk in college athletes

### Ricardo López García, José Omar Lagunes Carrasco, Rubén Ramírez Nava, Ricardo Navarro Orocio

#### Faculty of Sports Organization, Autonomous University of Nuevo Leon, Mexico

##### **Correspondence:** Ricardo López García: ricardo.lopezgr@uanl.edu.mx

**Introduction**: The association between bone quality and obesity have been investigated. Bone diameter could be a variable that influences body mass and the tendency to overweight and obesity. The aim of this study was to associate bone diameters with health risk variables such as BMI and abdominal perimeter.

**Materials and methods:** A descriptive study was carried out with a total of 62 men (21.13 ± 2.07 years of age) and 66 women (20.50 ± 1.76 years of age) university level athletes. Anthropometric measurements were taken using the ISAK protocol, calculating bone diameters (biacromial, biiliocrestal, femur and humerus), body weight, height and abdominal perimeter. The statistical program SPSS (version 26) was used, with Pearson correlation analysis.

**Results:** high correlations of the biilocrestal diameter and femur with BMI and abdominal perimeter were found (p ≤ 0.001), and moderate correlations between the biacromial diameter and humerus with BMI and abdominal perimeter (p ≤ 0.000).

**Conclusions:** With these findings we can indicate that the wider the bone diameter, the greater the tendency to an elevated body mass hence a higher BMI, as well as the increased abdominal area. However, the increase in BMI is often reflected in sports in which a considerable increase in muscle mass is required, although we must consider that sometimes this increase may be accompanied by abdominal fat. Therefore, we must take the results with caution and interpret them in the best way.

## A4 Analysis of heart rate variability during an 8-week training program in badminton athletes

### Mariela Flores-Cruz, José Miguel Herrera-Chávez, Rosa María Cruz-Castruita, Marina Medina Corrales

#### Facultad de Organización Deportiva, Universidad Autónoma de Nuevo León, Nuevo León, México

##### **Correspondence:** Marina Medina Corrales, marina.medinacr@uanl.edu.mx

**Background:** In the field of sport, the analysis of heart rate variability (HRV) is a tool of great contribution, one of the most used variables being the square root of the mean of the sum of the squares of the successive differences of the RR intervals (RMSSD) because it provides information on parasympathetic behavior and adaptation to workloads. Objective: To analyze the RMSSD values of youth badminton players during eight weeks of training.

**Materials and methods:** Ten badminton players (5 men and 5 women) from the State of Nuevo León, Mexico, belonging to the U-17, U-19, and Elite categories were part of the study. The measurements were made over a period of 8 weeks. The baseline HRV measurement was performed with the Firstbeat equipment for 5 minutes in the supine position. The records were analyzed with the Kubios HRV Standard software by previously inspecting each shot to detect the possible presence of irregularities. The individual values of the RMSSD were obtained by processing the information with the SPSS statistical package for the descriptive statistical analysis of each week, as well as differentiating by gender.

**Results:** Minimum values were found at the beginning of the period of 89.58 ms and 49.63 ms, as well as final values of 65.18 ms and 69.68 ms for men and women respectively, which indicates a lower recovery in the case of men at the end of the training period compared to women. It is observed that subjects 2 and 5 are those with the highest and lowest values (139.76 ± 29.77 and 33.48± 14.22) respectively throughout the 8 weeks.

**Conclusions:** Because workloads are changing during a training period, the response and adaptation to it have different behaviors, so it is important to have adequate and individualized monitoring to have improvements in sports performance.

## A5 An online home exercise program changes lower body strength, nutritional status but not depression scores during the COVID-19 in Mexican elderly

### Ermilo Canton Martinez^1^, Iván Rentería^1^, Patricia Concepción García-Suarez^1, 2,^ Gabriela Gutiérrez-Salmeán^3^, Juan Pablo Machado Parra^1^ and Alberto Jiménez-Maldonado^1^

#### ^1^ Facultad de Deportes Campus Ensenada, Universidad Autónoma de Baja California. Ensenada, México; ^2^ Department of Health, Sport and Exercise Sciences, The University of Kansas, USA; ^3^ Centro de Investigación en Ciencias de la Salud, Facultad de Ciencias de la Salud. Universidad Anáhuac México, Mexico

##### **Correspondence:** Alberto Jiménez-Maldonado, jimenez.alberto86@uabc.edu.mx

**Background:** Although the lockdown is considered one of the main strategies to reduce SARS-CoV2 transmission thus COVID-19 increasing rates, social isolation has also favoured sedentary behavior, sleep disorders, and unhealthy dietary habits in the elderly. Consequently, the practice of home-based physical exercise has been widely recommended to enhance health status during the COVID-19 quarantine. The current study was focused to assess the lower body strength, to screen nutritional status, and to determine depression levels in participants of a long-term online-guided exercise program for older adults during the COVID-19 quarantine

**Materials and methods:** Eleven participants (age: 63 ± 4.7 yrs.) carried out an online home exercise training program that included both aerobic and strength exercises comprising36 exercise sessions of 60 min performed 3 times/week, a withdrawal period (2 months). The 30-sec chair stand test was employed to evaluate lower body strength, while the walking speed was determined with the 4-meter walk test. To screen nutritional status, the short-form mini-nutritional assessment (MNA-SF) was used. Finally, depression levels were determined with the geriatric depression scale (GDS).

**Results:** Lower body strength was significantly improved with the exercise program. Likewise, the nutritional status screening scores increased. However, the online exercise program did not modify the depression score in older adults.

**Conclusions:** The findings of the current study showed that the exercise performed at home without specialized exercise equipment during the COVID-19 quarantine is an efficient stimulus to improve lower limbs strength in elderly people. Additionally, the data highlight physical exercise as a complementary factor to improve the nutritional status.

## A6 Anxiety symptoms in older adults during the COVID-19 pandemic. Intervention of physical exercise through social networks

### Manuel Octavio López-Camacho^1^, Arturo Alonso Cuevas López^1^, Rosa María Cruz-Castruita^2^

#### ^1^Departamento de Ciencias Sociales y Humanidades, Universidad Autónoma de Occidente, Sinaloa, México; ^2^ Facultad de Organización Deportiva, Universidad Autónoma de Nuevo León, Nuevo León, México

##### **Correspondence:** Manuel Octavio López-Camacho, tayo_080386@hotmail.com

**Background:** A serious problem derived from the COVID-19 pandemic was the increase in anxiety symptoms caused by the social restrictions imposed by the health agencies of our country, with the older adults being one of the most vulnerable groups in the face of these conditions. The use of information and communication technologies (ICT) was of great help in helping to combat health problems caused by social isolation and lack of physical activity (PA). The World Health Organization (WHO; 2020) considers that in the face of a pandemic, regular physical activity is necessary to improve mental health. The objective was to evaluate the anxiety symptoms of older adults in northern Mexico during the period of social confinement as a result of the COVID-19 pandemic and to present a proposal for physical exercise through social networks.

**Materials and methods:** It was a cross-sectional study, the data were collected through the Beck Anxiety Inventory (BAI), the sample was 20 older adults aged 60 to 76 years, the sampling was non-probabilistic through a snowball.

**Results:** 35% of older adults have minimal anxiety symptoms, 41% mild anxiety, 12% moderate anxiety and 12% severe anxiety. A physical exercise proposal was applied through social networks which lasted 16 weeks, working with a frequency of 3 days per week with a duration of 60 minutes per session, divided into warm-up, core part and relaxation, the Sessions were delivered through Whatsapp and Facebook, the participants were constantly monitored by members of the research team.

**Conclusions:** The high prevalence of older adults with symptoms of moderate and severe anxiety requires health promoters to address the problem, therefore the need arises to create options for non-pharmacological interventions through the use of ICT to follow the indications of restriction society in the face of a pandemic.

## A7 Assessment of energy expenditure and enjoyment during an exergame session in overweight and obese young adults

### Paulina B. Moreno Carbajal^1^, Norma E. Corrales Camargo^1^, Luis M. Gómez-Miranda^1^, Roberto Espinoza-Gutiérrez^1^, Juan J. Calleja-Núñez^1^, Bryan Montero-Herrera^2^, Jorge A. Aburto-Corona ^1^

#### ^1^ Sports Faculty, Autonomous University of Baja California, BC, México; ^2^ Physical Education School, University of Costa Rica, San Jose, Costa Rica

##### **Correspondence:** Jorge A. Aburto-Corona jorge.aburto@uabc.edu.mx

**Background:** The objective of this study is to determine the level of enjoyment and the amount of kilocalories (kcal) in an hour of exergame using the Nintendo Ring Fit Adventure in people with overweight and obesity.

**Materials and methods:** Participants: Thirteen male sedentary subjects (22.1 years; 177 cm in height; 104.8 kg in weight; VO^2^max 33.8 mL/kg/min) with overweight and obesity has been recruited (BMI 33.2; body fat 32.1%; muscle mass 39.5 kg).Participants attended in two occasions to the Human Motor Bioscience Laboratory. On the first visit, body measurements (i.e., height, weight, and body composition) were taken with a bioimpedance analysis. Next, a progressive loading protocol on a treadmill was carried on to determine the VO^2^max by indirect calorimetry. In the second session, participants performed 60 minutes of exergame with the Ring Fit Adventure. The exercise protocol was performed connected to the metabolic cart during the entire visit, requesting an *ad libitum* effort during the session. Once the time was over, the level of enjoyment of physical activity was determined using the 16-item PACES questionnaire.

**Results:** The preliminary analysis demonstrated that participants metabolized 6.4 kcal/min using the exergame Ring Fit Adventure, exercising at 68% (131.3 ± 14.4 bpm) of their HR_max_. Through the PACES questionnaire, a high percentage of enjoyment with this video game (88.8%) was reported.

**Conclusions:** The main finding of the study is that exercising with the Ring Fit Adventure (6.4 kcal/min) elicits similar caloric expenditure compared to continuous exercise training of moderate intensity (7.5 kcal/min). *Exergames* produce greater enjoyment and adherence to exercise compared to MICT and HIIT in people with overweight and obesity.

## A8 Leger test as a specific protocol to estimate the maximum oxygen consumption in the Urban Search and Rescue division of the fire department of Tijuana, Mexico

### Roberto Espinoza-Gutiérrez^1,2^, Eva Isabel Torres-Loma^1^, Michelle Yazmín Moroyoqui-Bolaños^1^, Juan José Calleja-Núñez^1^, Jorge Alberto Aburto-Corona^1,2^, Luis Mario Gómez-Miranda^1,2^, Elena Cecilia Guzmán-Gutiérrez^1^

#### ^1^ Sports Faculty, Autonomous University of Baja California, Tijuana, México; ^2^ Research Group UABC-CA-341 in “Physical Performance and Health”, Autonomous University of Baja California, Tijuana, Mexico

##### **Correspondence:** Roberto Espinoza-Gutiérrez, espinoza.roberto@uabc.edu.mx

**Background:** Fighting fires is a dangerous job that requires optimal fitness. Suboptimal physical fitness is detrimental and can lead some firefighters to exceed safe health limits at work. It has been documented that the principal cause of death in firefighters is sudden cardiac death. Therefore, the objective of this study is to identify the most appropriate field test to estimate the Maximum Oxygen Consumption (VO2max) in elements of the Urban Search and Rescue (USAR) group of the fire department of Tijuana, Mexico.

**Materials and methods:** Observational, descriptive and comparative study. Twenty-one USAR firefighters, members of the Tijuana Fire Department participated. The Cooper and the Leger tests were applied (one week apart). Maximum heart rate estimated and maximum post-exercise heart rate were recorded for each test (polar FT1). The VO2max of each subject were estimated. The Shapiro-Wilk test were used to determine the normality of the data. To determine if there were significant differences between the Cooper and Leger tests, a t-Student test was developed for independent samples.

**Results:** Significant difference (p=0.024) were found between the estimated maximum heart rate [184.9±4.5 beats per minute (bpm)] and the maximum post-exercise heart rate (177.1±15.6 bpm) in the Cooper test, but not in Leger test (184.9±4.5 bpm vs 185.5±10.3 bpm, respectively, p=0.784). In the same way, differences (p=0.008) in VO2max were observed between Cooper test (35.0±8.3 ml/kg/min) and Leger tests (38.2±5.6 ml/kg/min).

**Conclusions:** The tests considered in this study caused different physical effort among the participants. Cooper did not show to be a test in which the subjects perform a maximum effort, while the Leger test seems to be appropriate to induce a maximum effort and estimate the VO2max in field tests in the participants of this study.

## A9 Effect of physical exercise on Natural Killer cells

### Estefania Quintana^1^, Judith Rordíguez^1^, Gerardo Espino^2^, Claudia Carrasco^1^

#### ^1^Autonomous University of Chihuahua, Faculty of Physical Culture Sciences, Chihuahua, Mexico; ^2^Autonomous University of Chihuahua, Faculty of Medicine and Biomedical Sciences, Chihuahua, Mexico

##### **Correspondence:** Estefania Quintana, esquintana@uach.mx

**Background:** Natural Killer cells participate in the body's first line of defense against pathogens, viruses or cancer cells and these cells of the innate immune system are the most sensitive to the stimulus of exercise. Increases in peripheral blood have been observed within minutes of starting physical exertion, however, the specific response of its subpopulations is not yet clear. Due to this, the objective of the present study was to evaluate the acute effect of a session of aerobic physical exercise at moderate intensity on Natural Killer (NK) cell populations in healthy women.

**Materials and methods:** A session of aerobic exercise of 30 minutes at moderate intensity was applied to 23 healthy women and a blood sample was taken before and immediately after the exercise session to analyze by flow cytometry the NK total cells (CD3^-^CD56^+^), their effector (CD56^bright^CD16^dim^) and cytotoxic (CD56^dim^CD16^bright^) populations.

**Results:** NK total cells showed an increase after the exercise session, however, the response of their populations and receptors seemed to be influenced by age, body mass index and physical condition of the participants.

**Conclusions:** The increase in the cytotoxic population, as well as the decrease in the effector population, could be explained by their migratory potential towards specific tissues: while the cytotoxic population has affinity for the vascular endothelium, by increasing blood flow due to exercise, reservoirs are stimulated of this population and increases in peripheral blood; on the other hand, the effector population has a migratory potential towards secondary lymphoid tissues, which could suggest that the behavior of these populations due to exercise increases immunosurveillance in the organism, which would contribute to the prevention or treatment of established pathologies where the immune system is involved.

## A10 Resistance training using time under tension improves the muscle quality and muscle mass in healthy-young women. A preliminary study

### Manuel Alejandro Ceseña Peralta^1^, Iván Rentería^1^, Barbara Moura Antunes^1^, Patricia C. García-Suárez^1, 2,^ Juan Pablo Machado-Parra^1^, Alberto Jiménez-Maldonado^1^

#### ^1^ Facultad de Deportes Campus Ensenada (Universidad Autónoma de Baja California. Ensenada, México); ^2^ Department of Health, Sport and Exercise Sciences (The University of Kansas, USA)

##### **Correspondence:** Alberto Jiménez-Maldonado, jimenez.alberto86@uabc.edu.mx

**Background:** Strength training considering time under tension (T.U.T.) is a method that has proven it´s feasibility on the enhancement of strength and muscle mass in male population. Contrary, the effects on variables related to strength and muscle mass in female population, have not been addressed. Therefore, this project aimed to assess the impact of a strength training model based on T.U.T in body composition and muscle strength in physically active women.

**Materials and methods:** Six healthy women (age: 21.6 ± 1.9 years, BMI = 26.4 ± 1.0 kg/m2) were recruited, whom participated in the study, for the experimental design of the project, the subjects were randomly divided into 3 groups: s: Control: Without intervention; Traditional training (execution time: 1.0.1 s); and T.U.T. training, (execution time 1.0.3 s). Both strength training protocols lasted 4 weeks with the workloads established at 75% of a maximum repetition (1RM). The maximum strength of lower and upper limbs was determined by mean values of the 1RM test. On the other hand, absolute power, relative power and execution speed in the flat bench press, barbell row, barbell squat and deadlift (30% RM) exercises were assessed with a linear position transducer (GymAware). Muscle mass was determined by the bioelectrical impedance method (InBody 770).

**Results:** The main results of the study indicate that T.U.T.-based strength training has positive effects on muscle mass and body composition, performing a lower volume of mobilized workload In addition to the latter, T.U.T. training does not seem to have a negative effect on power and maximum strength in physically active women.

**Conclusions:** This results highlight the importance of T.U.T.- based strength training for coaches or trainers, since it involves variables with a high application in the sports training methodology.

## A11 Effects of a 16-session high intensity interval training program on body composition in sedentary subjects

### Luis Mario Gómez-Miranda^1,2^, Melinna Ortiz-Ortiz^1^, América Espinosa-Lezama^1^, Guadalupe Martinez-Raya^1^, Jorge A. Aburto-Corona^1,2^, Roberto Espinoza-Gutiérrez^1,2^, Juan J. Calleja-Núñez^1^, Marco A. Hernández-Lepe^3^, Diego A. Bonilla^4,5,6,7^

#### ^1^ Sports Faculty, Autonomous University of Baja California, Tijuana, México; ^2^ Research Group UABC-CA-341 in “Physical Performance and Health”, Autonomous University of Baja California, Tijuana, Mexico; ^3^ Medical and Psychology School, Autonomous University of Baja California, Tijuana México; ^4^ Research Division, Dynamical Business & Science Society – DBSS International SAS, Bogotá 110311, Colombia; ^5^ Research Group in Physical Activity, Sports and Health Sciences—GICAFS, Universidad de Córdoba, Montería 230002, Colombia; ^6^ Research Group in Biochemistry and Molecular Biology, Faculty of Sciences and Education, Universidad Distrital Francisco José de Caldas, 110311, Bogotá, Colombia; ^7^ Sport Genomics Research Group, Department of Genetics, Physical Anthropology and Animal Physiology, Faculty of Science and Technology, University of the Basque Country (UPV/EHU), 48940 Leioa, Spain;

##### **Correspondence:** Luis Mario Gómez-Miranda, lgomez8@uabc.edu.mx

**Background:** There are different ways to program the high intensity interval training (HIIT). The final velocity achieved during the 30−15 Intermittent Fitness Test (V_IFT_) has been reported as appropriate and reliable for team sports. However, less is reported in sedentary individuals of both sexes. The aim of this study was to evaluate the effect of a V_IFT_-based HIIT program on body composition in sedentary adults.

**Materials and methods:** This was an open-labeled repeated-measure study with a 16-session treadmill HIIT program (three sessions per week). The 30-15_IFT_ was applied in all participants and V_IFT_ was recorded. Warm-up consisted of a three-minute jogging at 40% of V_IFT_. The participants performed five high-intensity (80% V_IFT_) intervals for one minute each followed by a one-minute low-intensity interval (50% V_IFT_) and finished with five minutes of recovery (40% V_IFT_). Stature, body mass (BM), muscle mass (MM), body fat percentage (%BF) and visceral fat area (VFA) were measured at three time points: baseline (M1), before session nine (M2), and after 16 sessions (M3).

**Results:** Twelve sedentary adults (6M; 6F; age = 32.9±7.7 years; stature = 168.2±8.7 cm; BM = 83.0±16.6 kg; MM = 30.2±6.9 kg; %BF = 35±5.3%; VFA = 136.8±39.7 cm^2^) participated in this study. No significant differences were found in BM between the three measurements (p=0.633). However, when comparing M1 with M3, significant differences were found on MM (30.2 ± 6.9 kg, 30.6 ± 6.9 kg; p=0.029), %BF (35.0 ± 5.3%, 33.3 ± 5.1%; p=0.009), and VFA (136.8 ± 39.7 cm^2^, 126.7 ± 42.7cm^2^; p=0.001).

**Conclusions:** Sixteen sessions of V_IFT_-based HIIT (with a duration of 17 minutes per session) might significantly increase MM and decrease the %BF and VFA in sedentary adults.

## A12 Association between thermoregulation and mood in older adults in different protocols of physical exercise

### Felipe Garavito^1^, Astolfo Romero^1,2^, Isabel Sanchez^1^, Felipe Abril^1^, Dara Terán^1^

#### ^1^ Cultura Física, Deporte y Recreación, Santo Tomás University, Bogotá-Colombia; ^2^ Military Physical Education Department, Escuela Militar de Cadetes, Bogotá-Colombia

##### **Correspondence:** Felipe Garavito, felipegaravito@usantotomas.edu.co

**Background:** It is estimated that the world population by 2050 will reach 22% of the older adult population. On the other hand, increases in body temperature, favor a good state of the brain, showing changes in the affection of people who exercise, reducing depressive states, anxiety, increasing the perception of energy

**Materials and methods:** 23 older adults were exposed to four protocols: Strength, Endurance, Coordination, and Flexibility with an expenditure between 250 and 300 Kcal, measurements were taken before and after of central and peripheral temperature, and affectivity (PANAS). A Mixed design ANOVA with posthoc adjustment by Bonferroni and a Pearson correlation analysis was performed on the output variables.

**Results:** Disgusted Affect decreases in Resistance pre (M=1.52 and SD=0.97) and post (M=1.24 and SD=0.66); Interested increases, in Strength pre (M=4.70 and SD=0.53) and post (M=4.90 and SD=0.28); and Flexibility pre (M=4.45 and SD=0.63) and post (M=4.70 and SD=0.43); Irritated decreases, in Resistance pre (M=1.47 and SD=0.87) and post (M=1.09 and SD=0.41); Fearful decreases in Coordination pre (M=1.52 and SD=0.80) and post (M=1.05 and SD=0.20) and Flexibility pre (M=1.54 and SD=0, 92) and post (M=1.09 and SD=0.28). On the other hand, negative correlations are found as the effect of exercise on peripheral temperature vs Negative Affect Coordination and Flexibility (R2=0.39) and core temperature on Strength (R2=0.59), and positive on Resistance (R2=0.48) on temperature central as well as peripheral. Positive correlations are found as the effect of exercise on core temperature vs Positive Affect on Coordination and Resistance (R2=0.456), and changes in peripheral temperature are negatively related to the Coordination protocol (R2=-0.74).

**Conclusions**: Changes in peripheral temperature do not represent changes in mood in older adults, however, increases or decreases in temperature do seem to affect mood responses during physical exercise depending on the type of exercise.

## A13 Salivary cortisol response and anxiety during competition in college indoor soccer players

### Oscar Eduardo Sánchez-Muñoz ^1^, Diana María García-Cardona ^1^, Carlos Alfonso Bustamante Gutiérrez ^1^, Patricia Landázuri ^2^

#### ^1^ Programa de Educación Física, Recreación y Deportes, Universidad del Quindío, Quindío, Colombia; ^2^ Programa de Medicina, Universidad del Quindío, Quindío, Colombia

##### **Correspondence:** Diana María García-Cardona, dmgarcia@uniquindio.edu.co

**Background:** The study aimed to determine the response of salivary cortisol and anxiety during competition in college futsal players.

**Materials and methods:** The research was quantitative, descriptive and longitudinal in which 10 university players from that team participated. As study variables there were determined anthropometric aspects, cortisol, state and trait anxiety. Information was collected on the qualifying phase (3 games) of a university competition.

**Results:** Among the main results, it can be seen that anxiety levels both in trait and in state are presented in a positive way (State anxiety +: Trait anxiety +), in the category called: Above the average that is equivalent to a location in the area that ranges from the 55th to the 75th percentile; Regarding cortisol, on average, the highest concentration was presented once the last game was over.

**Conclusions:** The gap in the preparation of the athlete in relation to the psychological aspects, can affect the performance of the players and thus prevent them from reaching the levels of competitive performance necessary to perform effectively in terms of their physical and technical capacity which, for purely mental reasons, can be affected.

## A14 Associations between reactive strength and VO_2_-related variables in elite endurance athletes: a cross-sectional correlational pilot study

### Andrés Rojas-Jaramillo^1,2^, Óscar M. Cardona^3^, Juan Zuñiga^2,4^, Jorge L. Petro^2,5^, Luis M. Gómez-Miranda^6^, Jeffrey R. Stout^7^, Richard B. Kreider^8^ and Diego A. Bonilla^2,5,9,10^

#### ^1^ Research Group in Physical Activity and Sports Sciences (GRICAFDE), Instituto Universitario de Educación Física y Deporte, Universidad de Antioquia, 050001 Medellín, Colombia; ^2^ Research Division, Dynamical Business & Science Society – DBSS International SAS, 110311 Bogotá, Colombia; ^3^ Centro de Investigación del Deporte Antioqueño (CINDA), Instituto Departamental de Deportes de Antioquia – INDEPORTES Antioquia, Medellín, Colombia; ^4^ CenRED - Centro de Rehabilitación, Entrenamiento y Desempeño Deportivo, Bogotá, Colombia; ^5^ Research Group in Physical Activity, Sports and Health Sciences (GICAFS), Universidad de Córdoba, 230002 Montería, Colombia; ^6^ Sports Faculty, Autonomous University of Baja California, Tijuana, México; ^7^ Physiology of Work and Exercise Response (POWER) Laboratory, Institute of Exercise Physiology and Rehabilitation Science, University of Central Florida, Orlando, FL 32816, USA; ^8^ Exercise & Sport Nutrition Laboratory, Human Clinical Research Facility, Texas A&M University, College Station, TX 77843, USA; ^9^ Research Group in Biochemistry and Molecular Biology, Faculty of Sciences and Education, Universidad Distrital Francisco José de Caldas, Bogotá 110311, Colombia; ^10^ Sport Genomics Research Group, Department of Genetics, Physical Anthropology and Animal Physiology, Faculty of Science and Technology, University of the Basque Country (UPV/EHU), 48940 Leioa, Spain

##### **Correspondence:** Andrés Rojas-Jaramillo, arojasj@unal.edu.co

**Background:** Endurance performance has been shown to be enhanced by strength training. Therefore, there is a potential relationship between power-related variables such as the countermovement jump (CMJ) and drop jump (DJ) with specific cardiorespiratory performance parameters such as maximal oxygen consumption (VO_2max_) and velocity at VO_2max_ (vVO_2max_). This study evaluated the correlation between reactive strength and VO_2_-related variables in elite long-distance runners.

**Materials and methods:** Seventeen young Colombian elite endurance athletes (8F; 9M; 18.5 [2.2] years; 54.4 [4.0] kg; 162.4 [5.3] cm; 20.6 [0.7] kg∙m^-2^) participated in this cross-sectional correlational pilot study. Muscle power was assessed with the CMJ and DJ tests using a dual force platform (ForceDecks, VALD performance) at 1000 Hz. Reactive strength was calculated as absolute values of jump height and as the flight time-to-contact time (FT:CT) ratio at CMJ and DJ. A graded exercise test protocol was used to determine VO_2max_ and vVO_2max_ using a gas exchange analyzer (Quark RMR, COSMED).

**Results:** Statistically significant (p<0.05) sex-dependent differences were found in all variables (higher values in men). The results of the correlation analysis are expressed as Pearson's *r* (95% CI); *P* value. Overall, CMJ height presented a statistically significant correlation with VO_2max_ (0.64 [0.24, 0.86]; *P* < 0.01) and vVO_2max_ (0.64 [0.23, 0.86]; *P* < 0.01). Conversely, FT:CT at CMJ showed non-significant correlation with VO_2max_ and vVO_2max_. DJ height also presented a statistically significant correlation with VO_2max_ (0.64 [0.24, 0.86]; *P* < 0.01) and vVO_2max_ (0.57 [0.13, 0.83]; *P* < 0.01). FT:CT at DJ showed statistically significant correlation with VO_2max_ (0.67 [0.29, 0.87]; *P* < 0.01) and vVO_2max_ (0.61 [0.18, 0.84]; *P* < 0.01). As expected, VO_2max_ and vVO_2max_ correlated significantly (0.75 [0.42, 0.91]; *P* < 0.01).

**Conclusions:** There was a strong, positive and significant correlation between CMJ and DJ height, and FT:CT at DJ with VO_2_-related variables. Reactive strength-related variables may be considered for performance assessment in elite endurance athletes.

